# Supplementary material for: Multi-trait multi-environment genomic prediction strategies for Miscanthus sacchariflorus
Source: Front Plant Sci. 2026 Jul 23;17:1835247. doi: 10.3389/fpls.2026.1835247 (PMC13443311; doi:10.3389/fpls.2026.1835247)
Supplement: Supplementary file 1 [file DataSheet1.pdf]

Supplementary Material

1 Supplementary Figures and Tables

1.1 Supplementary Figures

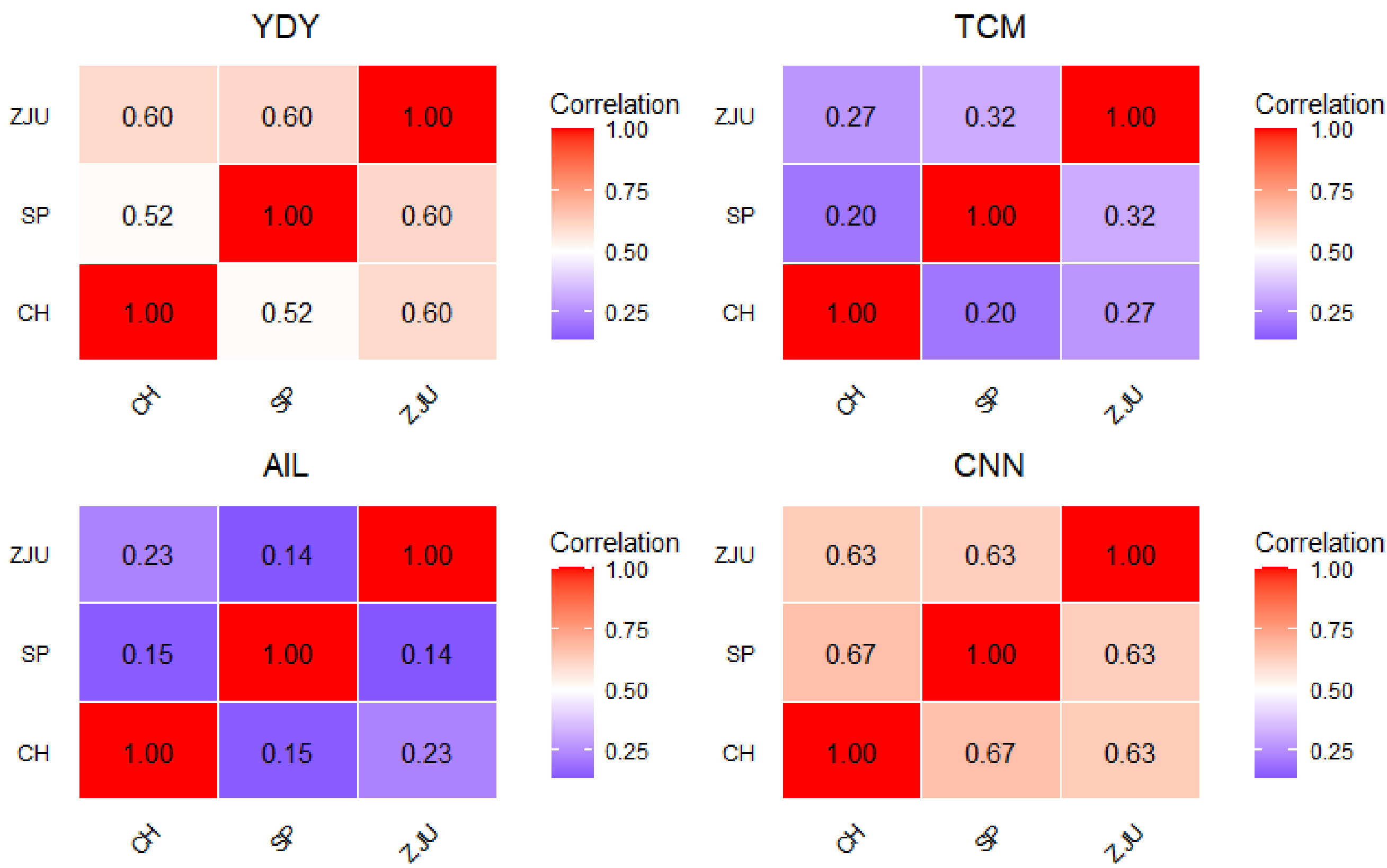

Figure S1. Phenotypic correlation between pairs of environments Sapporo, Japan by Hokkaido University (HU); Urbana, Illinois by the University of Illinois (UI); Chuncheon, South Korea by Kangwon National University (KNU) and Zhuji, China by Zhejiang University (ZJU) for a *Miscanthus sacchariflorus* population comprising 336 genotypes scored for biomass yield YDY, total culm number TCM, average internode length AIL, and culm node number CNN.
